# Neurological Consequences of COVID-19: A Systematic Review of the Pandemic’s Impact on Neurology Training

**DOI:** 10.3390/brainsci13081188

**Published:** 2023-08-10

**Authors:** Tommaso Ercoli, Francesco Barbato, Alessandro Bombaci, Luca Cuffaro, Francesco Di Lorenzo, Francesco Iodice, Michele Romoli, Paolo Solla, Giovanni Defazio

**Affiliations:** 1 Neurological Unit, AOU Sassari, University of Sassari, 07100 Sassari, SS, Italy; paolo.solla@aouss.it; 2 IRCCS San Raffaele Cassino, 03043 Cassino, FR, Italy; francescobarbato.88@hotmail.it; 3 “Rita Montalcini” Department of Neurology, University of Turin, 10124 Turin, TO, Italy; ale.bombaci@gmail.com; 4 Department of Medicine and Surgery, University of Milano-Bicocca, 20126 Milan, MI, Italy; cuffaro.luca@gmail.com; 5 Noninvasive Brain Stimulation Unit, Scientific Institute for Research, Hospitalisation and Health Care Santa Lucia Foundation, 00179 Rome, RM, Italy; f.dilorenzo@hsantalucia.it; 6 Department of Neuroscience and Neurorehabilitation, IRCCS San Raffaele Pisana, 00163 Rome, RM, Italy; franc.iodice@gmail.com; 7 Neurology and Stroke Unit, “Maurizio Bufalini” Hospital, 47521 Cesena, FC, Italy; romoli.mic@gmail.com; 8 Department of Clinical, Surgical and Experimental Sciences, University of Sassari, 07100 Sassari, SS, Italy; 9 Department of Translational Biomedicine and Neuroscience, University of Bari, 70121 Bari, BA, Italy; giovanni.defazio@uniba.it

**Keywords:** COVID-19, neurology, training, education, residents

## Abstract

The COVID-19 pandemic had a significant impact on neurology training programs, leading to disruptions and changes that may have long-term implications for neurological education. The objective of this study was to investigate the impact of COVID-19 on neurological training programs, collecting available data relating to residents’ experience worldwide. We performed a systematic search of the literature published on PubMed from January 2020 to March 2023, including studies referring to quantitative analysis of residents’/trainees’ perspectives. Specifically, we included studies that examined how the pandemic has affected clinical and research activities, the use of telemedicine, the delivery of education and the psychological status of residents. Of the 95460 studies identified through database searching, 12 studies met the full criteria and underwent data extraction. In conclusion, the COVID-19 pandemic has had significant impacts on neurology training programs, highlighting the need for resilience and flexibility in medical education. Future research should focus on the long-term outcomes of these adaptations in the quality of neurology education and patient care.

## 1. Introduction

The COVID-19 pandemic has presented an unprecedented challenge to healthcare systems worldwide [1]. Since the first case was reported in December 2019, the virus has rapidly spread and affected millions of people [2]. Indeed, COVID-19 has highlighted the importance of preparedness and flexibility in the face of unexpected events, particularly in the context of neurological diseases [3]. Over the last three years, several neurological symptoms have been associated with SARS-CoV-2 infection [4], highlighting the need to better understand the pathophysiologic mechanisms of this putative relationship [5]. The global crisis has dramatically changed with the spread of vaccines all over the world [6]; however, the implications and consequences of the outbreak in the healthcare system are still important points to investigate.

Within this context, the pandemic also had a significant impact on neurology training programs, leading to disruptions and changes that may have long-term implications for neurological education [7]. Indeed, neurology residents have experienced important and challenging modifications in their clinical practice and in the organization of their neurology residency programs and methods of education [8,9,10].

During the first part of the outbreak, hospitals rapidly shifted their mission to the management of COVID-19 patients, and many residents participated directly in this process, working in COVID-19 units [11]. Use of telemedicine became prominent from the beginning of the outbreak, and the delivery of education was deeply affected by social restrictions [10,12]. 

The aim of this systematic review is to investigate the impact of COVID-19 on neurological training programs, collecting available data on residents’ experience worldwide. To our knowledge, this is the first review that systematically addresses the consequences of COVID-19 on neurology residency programs. 

## 2. Methods

We performed a systematic search of the literature published on PubMed from January 2020 to March 2023 using the following searching string: (((COVID-19) AND (neurology)) AND (training)) OR (residency). We conducted a systematic review (not registered) following the Preferred Reporting Items for Systematic Reviews and Meta-Analyses (PRISMA) guidelines [13]. Only studies referring to quantitative analysis of residents’/trainees’ perspectives and published in English were considered. After duplicates were removed, the title, abstract and keywords of retrieved publications were screened by one author, and irrelevant studies were excluded. The reference list of each selected article was checked to screen for additional studies possibly worth including but which were not captured by the original search method.

## 3. Results

**Figure 1:**
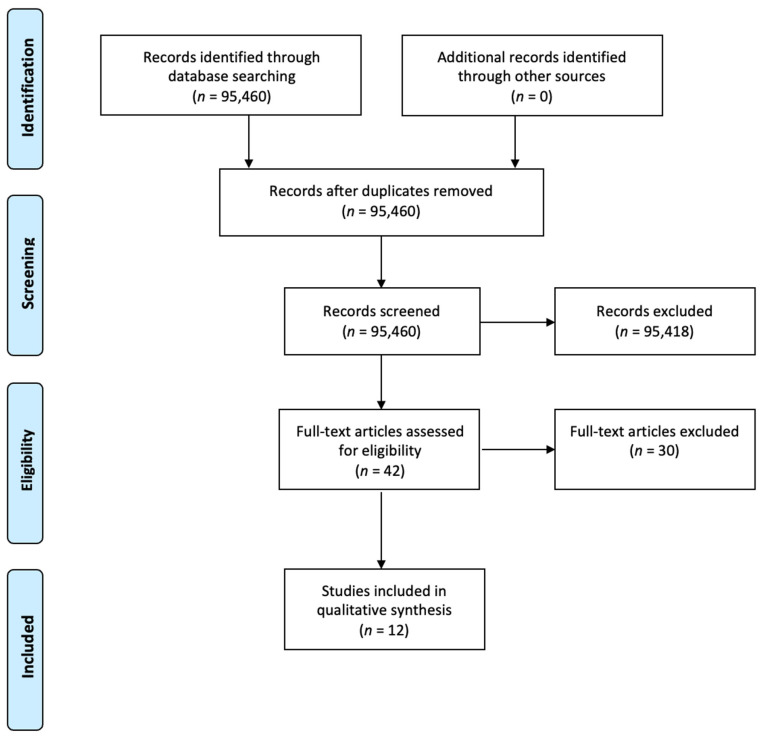
Study flow chart phases of the systematic review according to the Preferred Reporting Items for Systematic Reviews and Meta-Analyses (PRISMA) flow diagram guidelines.

Of the 95,460 studies identified through database searching, 95,418 records were excluded because the abstract or the title did not fulfil the inclusion criteria for this review (i.e., were not studies about neurology residents’/trainees’ perspectives on the impact of COVID-19 on their residency or quantitative analysis of these findings). Forty-two full papers were assessed for eligibility, and, eventually, only 12 studies met the aforementioned full criteria and underwent data extraction (Figure 1) [14,15,16,17,18,19,20,21,22,23,24,25]. Information related to year of publication, study design, sample size, study population, time and country of assessment is reported in Table 1.

Full-text articles were then independently reviewed by two authors for eligibility and included if they comprised quantitative analysis of residents’/trainees’ perspectives with specific reference to clinical and research activities, the use of telemedicine, the delivery of education and the psychological status of the residents engaged in neurology. Disagreements in study inclusion were resolved through iterative discussions with the other authors until consensus was achieved. Data were framed into a narrative review.

### 3.1. Impact on Clinical Activities

The Italian study by Di Lorenzo and colleagues found that the 97% of the Italian residents who completed the survey changed their clinical routine because of the outbreak [15]. The majority reduced their work shifts, mainly working remotely, while only 26% of the respondents increased their workload. The latter finding was observed in the most affected Italian territories hit by COVID-19 [15]. Similar findings were confirmed in the survey by Abati and Costamagna [16], who observed that the vast majority (87.3%) of the Italian residents reported a substantial reduction in their neurologic clinical activities. Moreover, 17.8% of the trainees were also recruited or volunteered for COVID-19 wards. In the study conducted by Farheen on US residents and fellows [18], trainees reported a variety of changes to their schedules in response to the pandemic. A significant portion of respondents reported a decrease in inpatient schedules (33.6%), while almost a third reported no change (29%), and a similar proportion reported an increase (28%). In the outpatient setting, more than half reported a reduced clinic schedule (56%), with about 11% reporting an increase and about one third (32%) reporting no change. Zeinali and other Iranian colleagues [21] reported that, at the beginning of the outbreak, all optional and non-urgent procedures were delayed. Following the pandemic’s onset, clinics were merged, and the daily number of active residents dropped from 15 trainees before COVID-19 to 6 active residents two months after the outbreak. In the study by Geronimo and colleagues from the Philippines, residents were reorganized into restricted teams and divided into non-COVID-19 and COVID-19 roles to prevent the spread of the virus. Since the workforce priority transitioned to managing the surge of COVID-19 patients, elective rotations for neuro-subspecialties were put on hold indefinitely [22]. In the work by Cuffaro and colleagues, which canvassed 227 Resident and Research Fellow Section (RRFS) members of the European Academy of Neurology (EAN) [23], the reduction in time spent with neurological patients during the pandemic was revealed to be a matter of concern for many. In terms of severity, 18% of respondents reported a severe reduction, while 31% experienced a moderate decrease in time spent with patients. A mild reduction was noted by 28% of participants, and 10% faced a very mild decrease. Interestingly, 13% of those surveyed claimed there was no reduction in time with neurological patients. Similarly, the decrease in supervision at work was also affected. A severe reduction in supervision (90–100%) was reported by 12% of respondents, and a moderate reduction (70–80%) was experienced by 10%. Mild (50–60%) and very mild (30–40%) reductions were noted by 19% and 18% of participants, respectively. Encouragingly, 21% of those surveyed reported no reduction (0–20%) in supervision at work during the pandemic [23]. In a study evaluating the impact of COVID-19 in tertiary care neurology centers in Pakistan, it was found that 69.7% of neurology trainees were assigned to work in COVID-19 isolation units, and 66.7% of residents/interns had their daily duty schedules converted to on-call schedules only [24].

### 3.2. Impact on Research Activities

According to the study by the young section of the Italian Society of Neurology [26], most of the canvassed residents reported reduced research activities [15]. However, for some residents, the reduction in clinical activities was associated with an increase in research activities conducted remotely, and, interestingly, 43% of residents revealed that they had sufficient facilities to continue their research at home [15]. Similar data were obtained by Abati and Costamagna [16], who stated that the majority of canvassed trainees reported a decrease in face-to-face research activity during the pandemic, mainly due to the partial/total closure of research laboratories, clinical trials suspension and the impossibility of enrolling new patients. On the other hand, just a few trainees reported that research activity increased or did not change. A study from the Philippines highlighted the interruption of individual research projects due to logistical limitations and a shift in research focus towards investigating the relationship between COVID-19 and neurologic symptoms/diseases (i.e., a nationwide study on the neurological manifestations of COVID-19 in the Philippines) [22]. The EAN survey showed that a striking 21% of respondents experienced a severe impact (90–100%) of the pandemic on resident research projects, while 18% reported a moderate effect (70–80%). Additionally, 17% of participants faced mild consequences (50–60%), and a smaller proportion, 6%, encountered very mild repercussions (30–40%). Interestingly, an equal number of respondents (6%) claimed that the pandemic had no effect (0–20%) on their research projects [23].

### 3.3. Telemedicine

Data from Italian residents showed that, during the COVID-19 pandemic, there was a wide use of telemedicine in Italy, especially in comparison with in the past, when this method was occasionally used only by 14% of neurology residents [15]. Due to the suspension of clinical activities across the United States, Gummerson and colleagues [14] wanted to test the effectiveness of telemedicine in a sample of medical students, residents and fellows in the field of neurology. This study found that the virtual clinical elective successfully increased students’ confidence in virtually obtaining a history and performing a telehealth neurological physical exam. In the study by Farheen et al., which canvassed US trainees [18], 91% of the respondents reported using telemedicine in both outpatient and inpatient settings (43.2%) and for both new and follow-up patients (78%). However, the study found that only 42% of respondents received training in telemedicine, including training on how to perform neurological examinations. The investigation conducted among neurology consultants and residents in Saudi Arabia by Hmoud and others [19] revealed that consultants demonstrated significantly higher confidence levels in conducting physical examinations virtually than neurology residents. Notably, the desire to continue providing virtual health services after the pandemic was higher among consultants than among residents. Similar findings were reported in a study of Norwegian neurologists by Kristoffersen and colleagues [20]; indeed, they found that virtual management of movement disorders was primarily handled by senior consultants, whereas no significant differences in the use of telemedicine were detected for other diseases (e.g., epilepsy, headache and multiple sclerosis). Similarly to other situations, Iranian colleagues [21] implemented a virtual follow-up procedure utilizing phone or internet communications, particularly for patients receiving immunosuppressive drugs, and, furthermore, a multidisciplinary remote team was built to expedite decision-making processes. In the Philippines as well, teleconsultation, in compliance with the local guidelines, was widely used by residents for the management of non-urgent cases [22]. Among the surveyed professionals of the EAN study by Cuffaro and others [23], 28% of the canvassed RRFS members reported receiving official authorization from local authorities; meanwhile, 20% indicated that their telemedicine practices were allowed by government or official authorities without receiving official medical codification. A considerable 56% of respondents used telemedicine on a voluntary basis, mostly depending on availability [23]. Interesting and quite different data emerged in the study by Kolikonda and colleagues in the US. Indeed, over a third of residents (37%) felt uneasy about the use of telemedicine, and 16% believed it delayed stroke assessments and hindered their independence. The majority was also unsure about making telemedicine a routine part of stroke evaluations post pandemic [25].

### 3.4. Change in the Delivery of Education

In the vast majority (92%) of the Italian centers examined by Di Lorenzo and others, both lessons and seminars were delivered on online platforms [15]. The survey by Abati and Costamagna [16] found that the majority (51.9%) of respondents reported interruption of educational activities, while virtual platforms were used for the delivery of educational activities in 30.4% of cases. In order to mitigate the impact of COVID-19 on educational effectiveness, Zeinali and colleagues reported that several activities transitioned to webinars. This virtual setting enabled collaboration with other neurology centers, and, notably, the frequency of educational sessions increased in the second month of the outbreak [21]. Similar strategies were adopted in the Philippines, where academic activities for neurology residents were shifted to blended online learning, and trainees were encouraged to attend local and international webinars [22]. A significant 54% of the RRFS members of the EAN reported that their classes were suspended and postponed, while 10% indicated that their classes were canceled but that they were provided with papers to read or topics to study [23]. About a quarter of those surveyed revealed that their classes were not suspended but transitioned to an online format. Interestingly, a very small proportion of participants (1%) stated that their classes were not suspended at all during the pandemic [23]. As in other countries, also in Pakistan; it was observed that 60.6% of regular teaching sessions designed for neurology trainees transitioned to digital platforms [24]. In a US study of a single stroke center, a survey showed that 45% of trainees agreed that digital consultations did not hinder learning or education in the field of stroke services. However, 45% of neurology residents felt that the quality of bedside instruction was adversely affected by digital consultations [25].

### 3.5. Psychological Implications

The Italian investigation by Di Lorenzo and others showed that psychological support for residents working during the outbreak was offered to a quarter of neurology trainees [15]. Although the study by Di Liberto and others [17] was not strictly focused on the impact of COVID-19 on neurology residents, it provided interesting information about neurology residents, junior neurologists and research fellows from Europe. Indeed, despite the 44% of the respondents who stated that their workload had grown due to the pandemic, the authors did not find a consequent increase in symptoms of burnout. On the other hand, the American survey by Farheen and others [18] revealed that a majority (75%) of respondents experienced moderate to very high levels of additional stress during the pandemic, and 33% of trainees reported difficulty obtaining childcare during the pandemic. Trainees’ mental health concerns were addressed by local institutions in the Philippines by the implementation of a “buddy” system. In this approach, consultants provided moral support to assigned neurology residents, assisting with both workload and personal issues [22]. In the EAN survey on RRFS members, nearly half (49%) of the respondents reported availability of psychological support at their hospital/university. However, it is important to note that for 18% of those surveyed, this assistance was offered on a voluntary basis by a psychiatrist or psychologist [23]. In the study evaluating the impact of the pandemic in Pakistan, it was found that at just 36.4% of the tertiary care neurology centers did the hospital administration provide mental health support services for healthcare providers [24].

## 4. Discussion

According to this systematic review, the COVID-19 pandemic has caused significant changes to neurology training programs worldwide. These modifications have affected various aspects of residency, including clinical and research activities, the implementation and use of telemedicine, the delivery of education and the psychological well-being of the residents.

The impact on clinical activities was reported to be substantial in almost all the studies included in this review. There has been a widespread decrease in time spent with neurological patients [15,16,18,23], leading to potential concerns about the effect this reduction might have on the quality of neurology training. Moreover, a specific focus of neurology residents on COVID-19 patient care was also reported in several countries [15,16,22,24], which may have detracted from specialized neurological education. 

Research activities were generally reported as having decreased during the outbreak [15,16,22,23], mainly due to restrictions on hospital/university access and to the reallocation of resources towards managing the pandemic emergency. However, some residents reported that they were able to maintain or even increase their research activities, potentially due to the opportunity to work from home with remote tools and the possibility to focus only on this task [15].

The use of telemedicine has been dramatically amplified during the pandemic. This sudden increase has demonstrated the potential utility of this resource but has also highlighted potential issues regarding the confidence and proficiency of residents in delivering virtual care [14,15,18,19,20,21,22,23,25]. Further training and support for residents in this area might be beneficial for the future considering the potential key role of telemedicine in healthcare delivery. The next generation of neurologists, who will practice in the post-COVID era, will be the first to experience the integration of web-based clinical management. Urgent attention is required to establish general rules of use for this approach and assess the limitations and potential risks associated with this form of healthcare delivery. In particular, limited physical examination, technology dependence, privacy concerns and restricted internet access are the major disadvantages of telemedicine to overcome in the near future [27,28]. Although patients’ perspectives on telemedicine have been studied only under COVID-19 restrictions, it seems that many patients were satisfied with remote consultations. Further studies are needed to better understand patients’ opinions about telehealth after the pandemic [29,30].

Online learning was widely use in several hospitals/universities for the delivery of neurology education [15,16,21,22,23,24]. Although this method was generally successful in residents’ opinion, further efforts might be necessary to optimize online education strategies and to guarantee the practical aspects of clinical neurology training. As far as medical education is concerned, the e-learning approach has increased interest in neurology among medical students, especially when interactive discussions, practice and feedback were offered [31,32].

Moreover, the pandemic also forced a shift from in-person conferences/meetings to virtual conferences. After initial hesitation about moving all material and presentations online, due mainly to technical reasons and also to the feeling of impersonality due to the nature of the virtual meeting, virtual conferences fulfilled the main mission of organizing high-quality congresses despite the restrictions of the impersonal format, as evidenced by the high registration numbers of attendees [33]. The geographical distribution of the participants of virtual meetings proved that virtual conferences have higher inclusivity, and so they represent an opportunity for wider participation of students and neurologists in training [33].

As far as psychological implications are concerned, the pandemic increased stress levels [15,17,18,22,23,24] among neurology trainees, a finding that calls for enhanced efforts to support the mental health of residents during such global crises. This may involve increased psychological support from hospitals and universities, as well as changes to work schedules and duties to prevent burnout.

Our findings are in line with a worldwide WHO survey of international neurological patient and scientific organizations that examined the disruption and mitigation of neurological services during the outbreak [34]. The authors of this study detected that many educational activities (60%) and residency/PhD study programs in all neurology-related fields (39%) were reorganized due to the pandemic [34]. Additionally, 44% of participants stated that neurology residents were engaged in managing COVID-19 patients from the first wave, either in general COVID-19 wards or neuro–COVID-19 units. Participants also indicated that the outbreak impacted neurology research in terms of both funding distribution and research endeavors [34].

This review had some limitations, including potential selection bias due to the inclusion criteria and the fact that some regions may be overrepresented in the data. Additionally, the studies included in this review were conducted at different points during the pandemic, which could have influenced the results due to changing circumstances and adaptations over time. Moreover, none of the studies evaluated the academic performance of neurological trainees.

In conclusion, the COVID-19 pandemic has had significant impacts on neurology training programs, highlighting the need for resilience and flexibility in medical education. The lessons learned during this crisis could inform future adaptations to residency programs, ensuring that trainees receive comprehensive and effective training even in the case of unprecedented challenges. Future research should focus on the long-term outcomes of these adaptations in the quality of neurology education and patient care. 

## Figures and Tables

**Table 1:** Information on the 12 studies included in the systematic review.

Reference Number	Author(s)	Year of Publication	Study Design	Time of the Assessment	Sample Size	Study Population	Country
[14]	Gummerson et al.	2021	Observational study (survey)	May/July 2020	351	Residents + medical students + pre-medical students + fellows + faculty	US + attendance all over the world
[15]	Di Lorenzo et al.	2021	Observational study (survey)	April/May 2020	254	Residents	Italy
[16]	Abati et al.	2020	Observational study (survey)	April 2020	79	Residents	Italy
[17]	Di Liberto et al.	2022	Observational study (survey)	September 2020/January 2021	332	Residents + junior neurologists + research fellows	Europe
[18]	Farheen et al.	2021	Observational study (survey)	June/August 2020	285	Residents and fellows	United States
[19]	Hmoud et al.	2023	Observational study (survey)	September/December 2021	67	Neurology practicing physicians (including residents)	Saudi Arabia
[20]	Kristoffersen et al.	2021	Observational study (survey)	April 2020	57	Neurology practicing physicians (including residents)	Norway
[21]	Zeinali et al.	2020	Observational study limited to one department	March/April 2020	N/A	Neurology practicing physicians (including residents)	Iran
[22]	Geronimo et al.	2022	Observational study limited to one department	2020	N/A	Residents	Philippines
[23]	Cuffaro et al.	2020	Observational study (survey)	From 29 April to 25 August 2020	227 (15%)	Residents + research fellows + young neurologist	Europe + other countries
[24]	Kanwar et al.	2021	Observational study (survey)	July 2020	33 out of 40 tertiary care neurology centers (83%) in Pakistan	An estimated 1300 healthcare workers (faculty, medical officers, trainees and nurses) working at these 33 participating centers.	Pakistan
[25]	Kolikonda et al.	2021	Observational study (survey)	May to June 2020	32	Residents + fellows + others	United States
